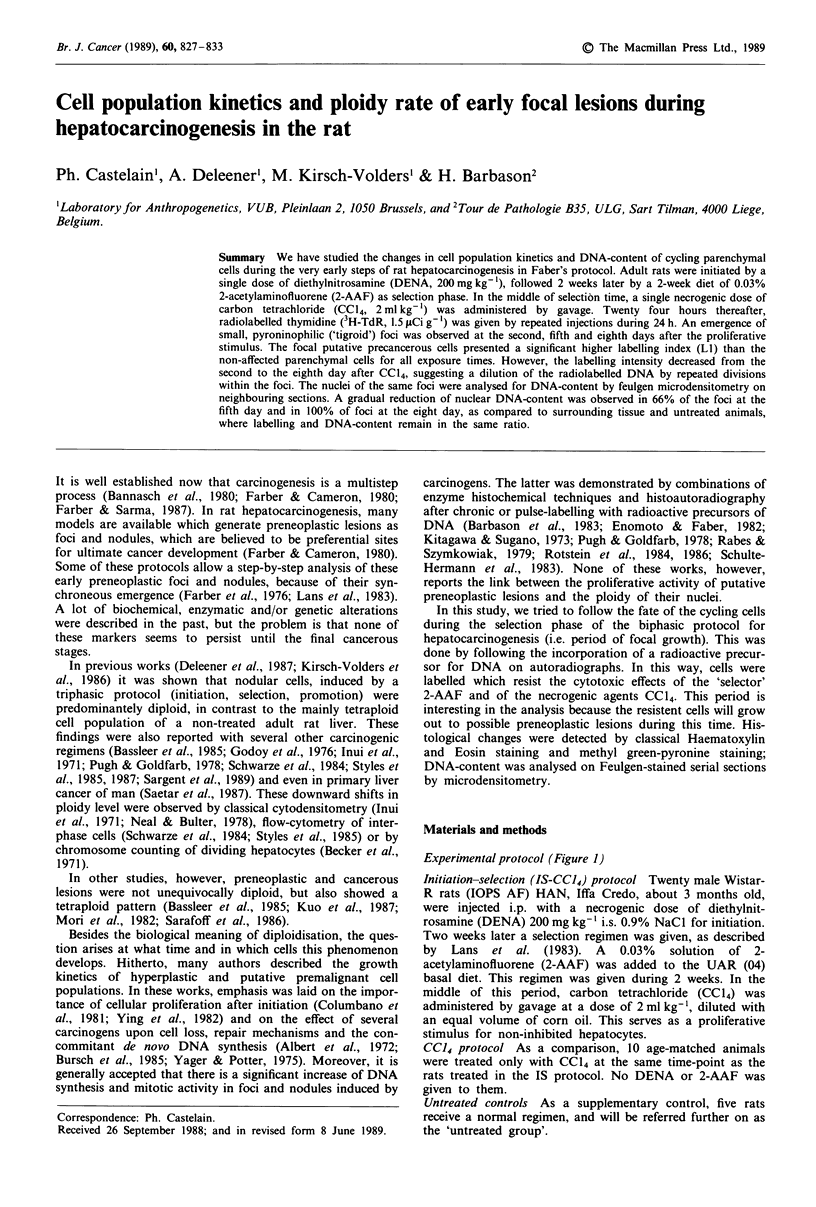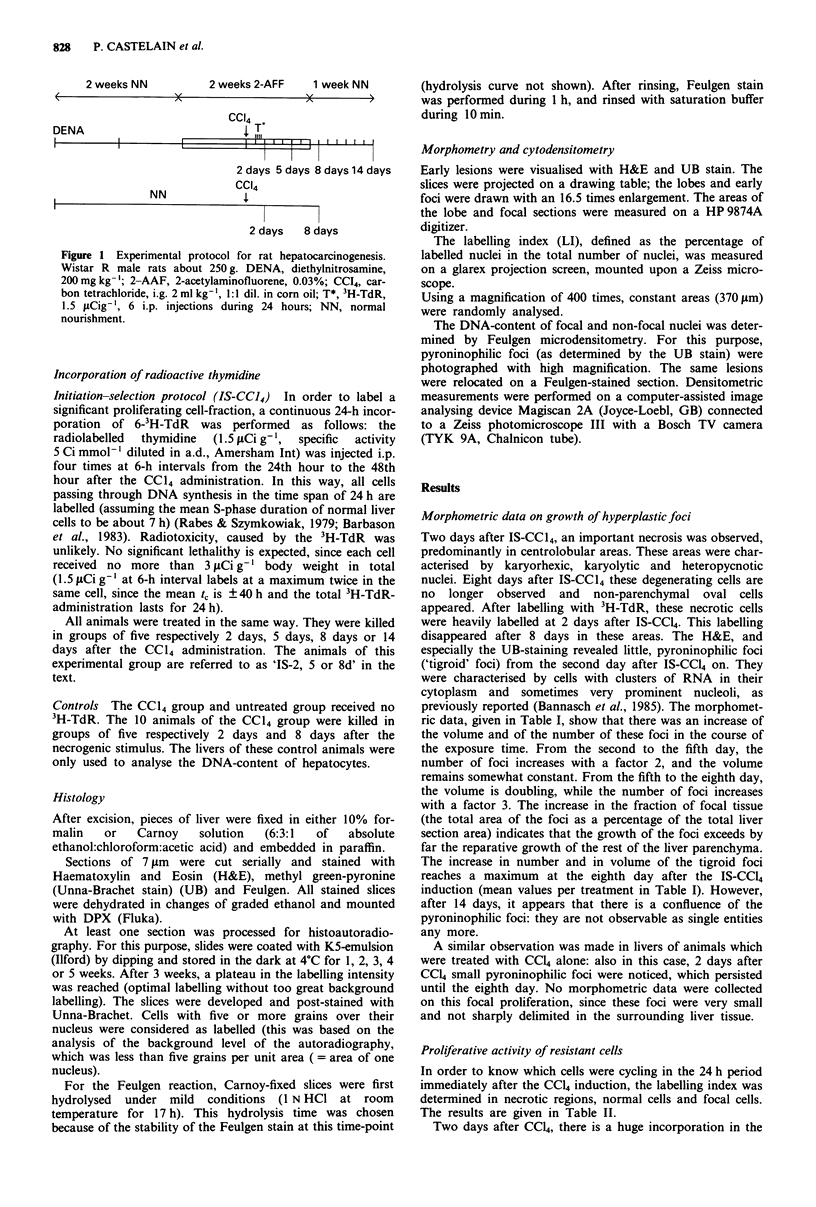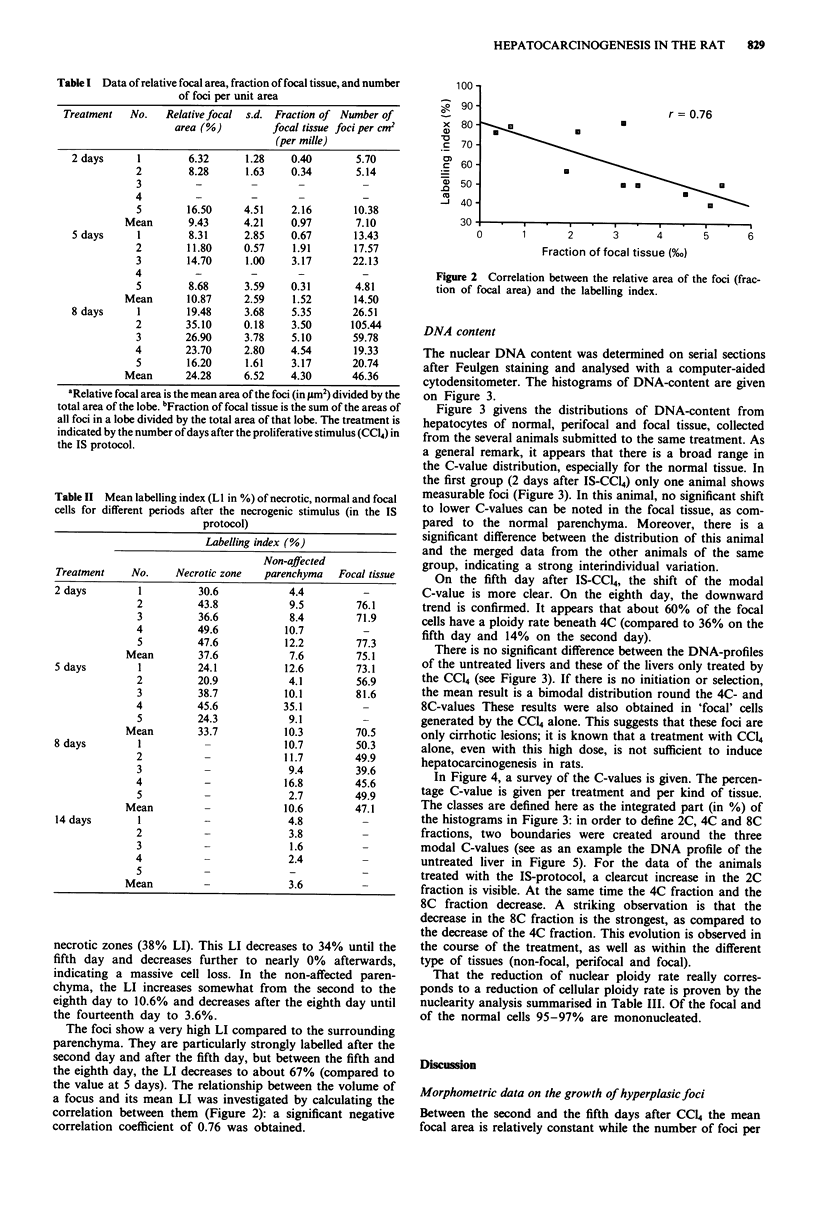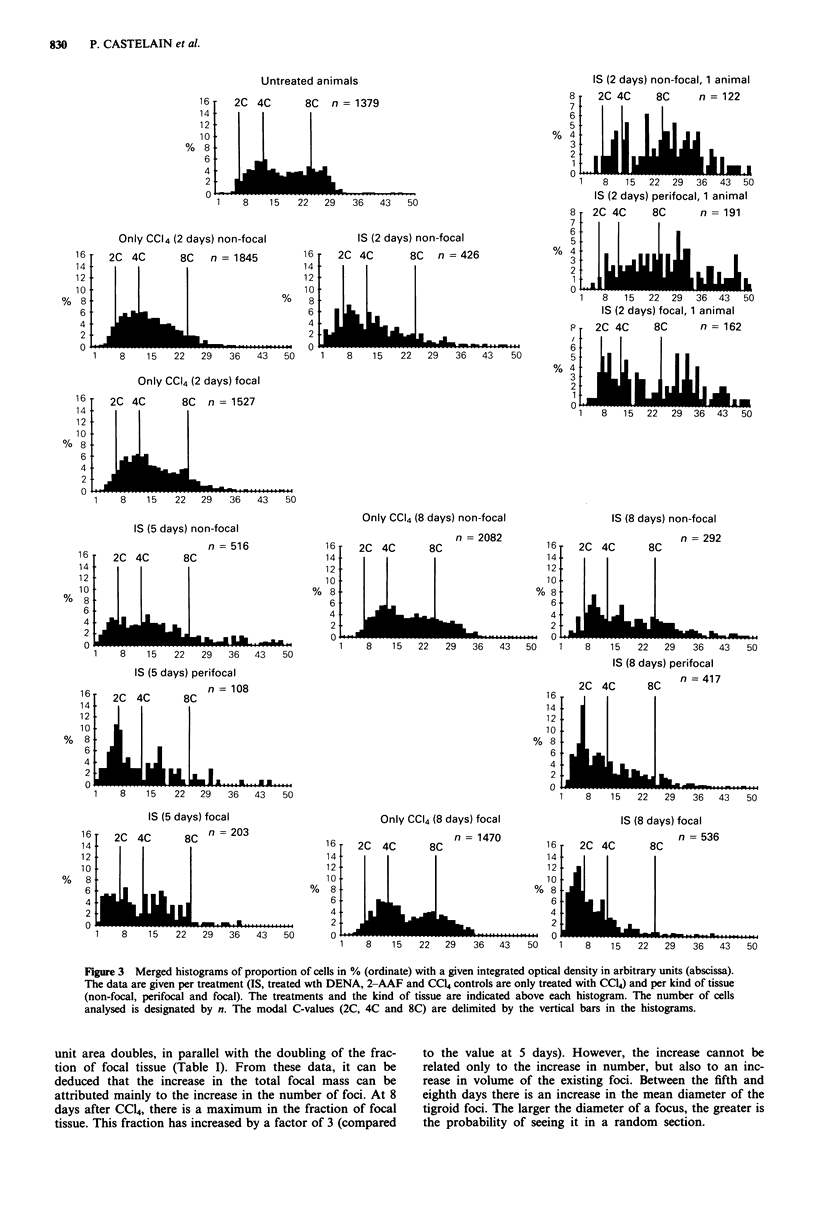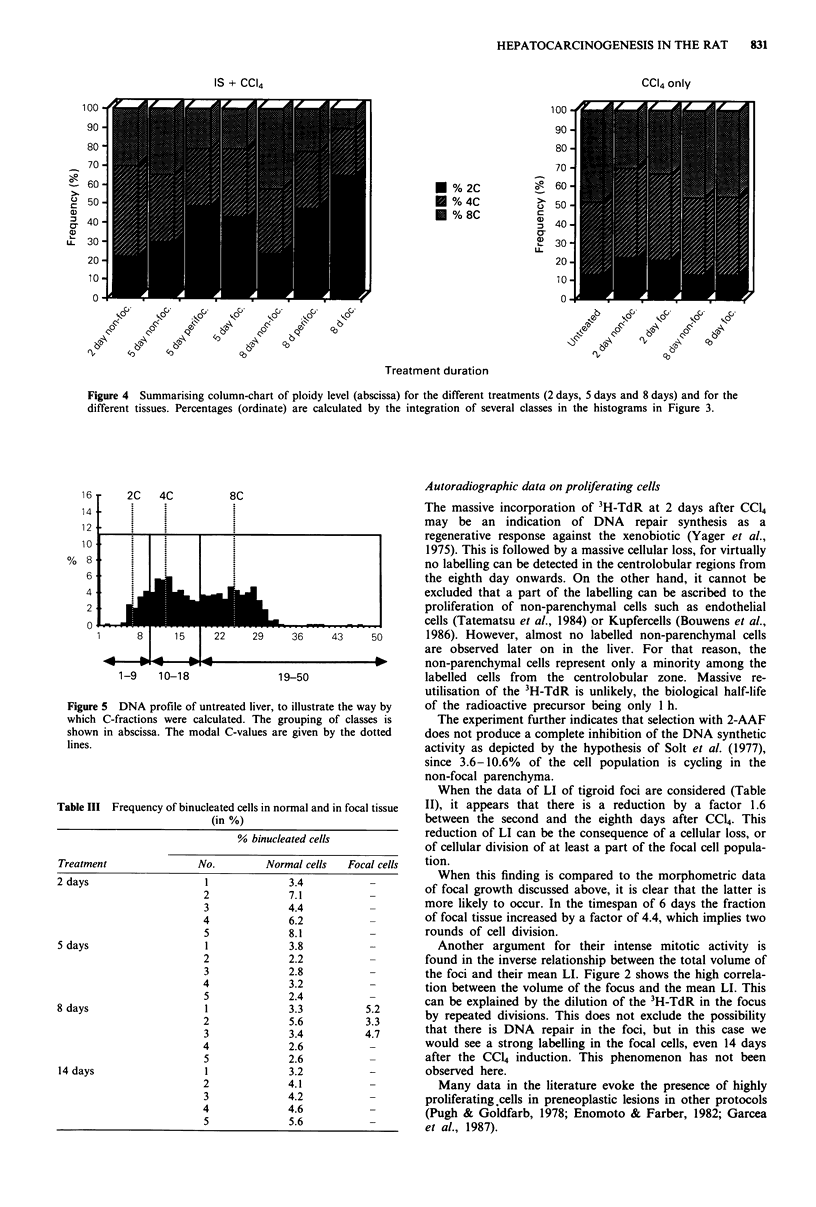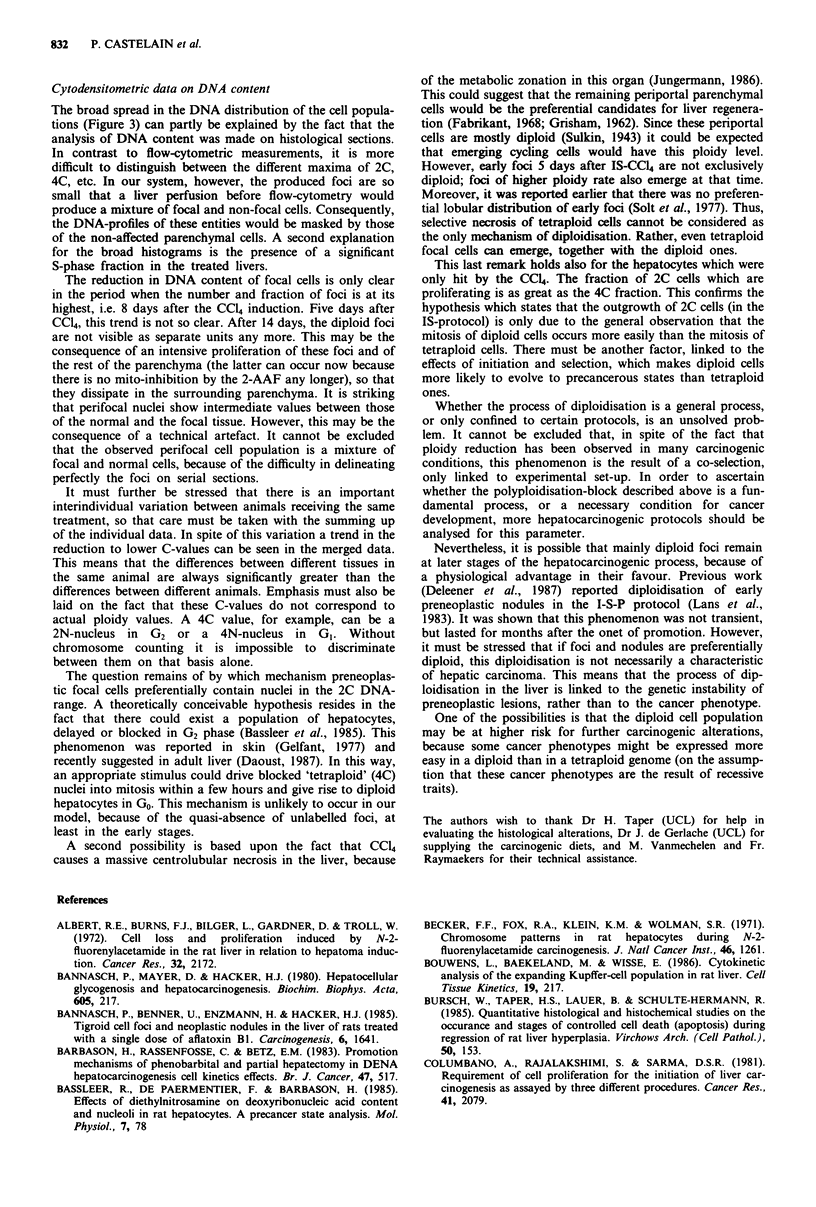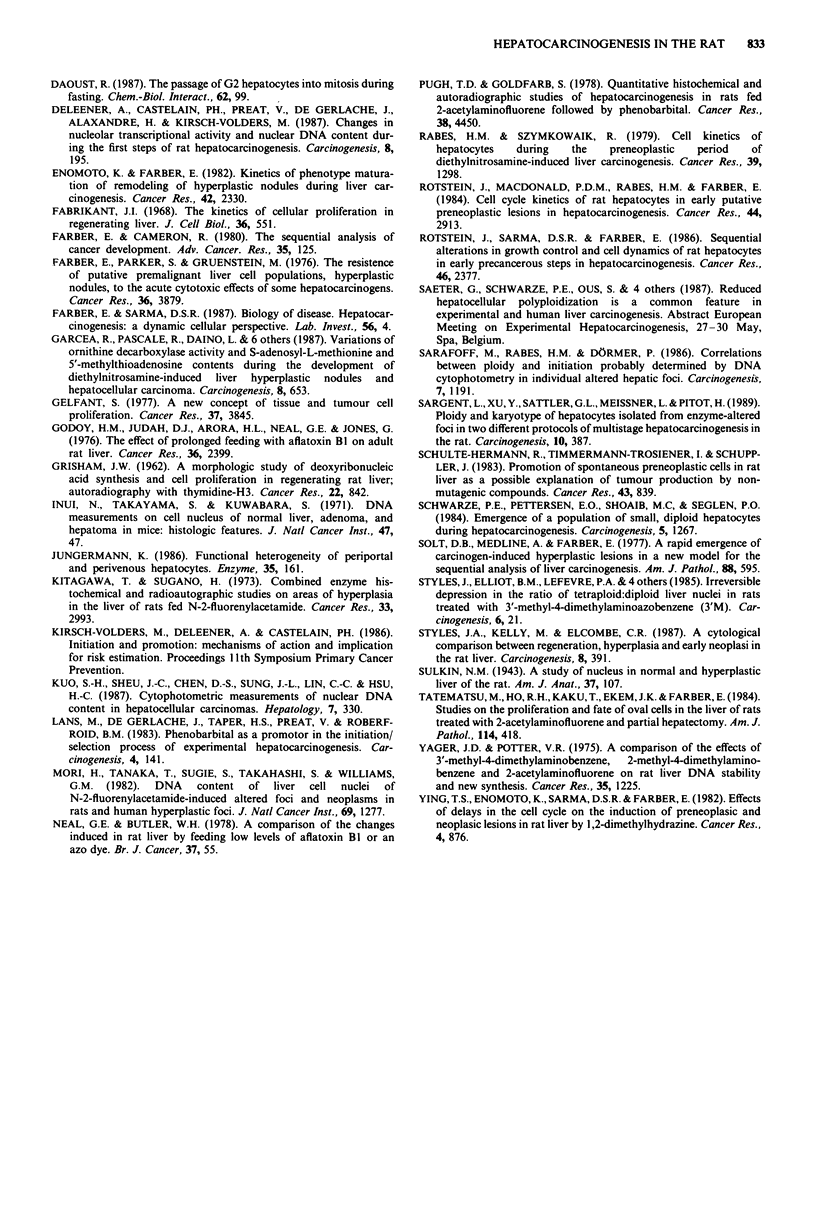# Cell population kinetics and ploidy rate of early focal lesions during hepatocarcinogenesis in the rat.

**DOI:** 10.1038/bjc.1989.374

**Published:** 1989-12

**Authors:** P. Castelain, A. Deleener, M. Kirsch-Volders, H. Barbason

**Affiliations:** Laboratory for Anthropogenetics, VUB, Brussels, Belgium.

## Abstract

We have studied the changes in cell population kinetics and DNA-content of cycling parenchymal cells during the very early steps of rat hepatocarcinogenesis in Faber's protocol. Adult rats were initiated by a single dose of diethylnitrosamine (DENA, 200 mg kg-1), followed 2 weeks later by a 2-week diet of 0.03% 2-acetylaminofluorene (2-AAF) as selection phase. In the middle of selection time, a single necrogenic dose of carbon tetrachloride (CCl4, 2 ml kg-1) was administered by gavage. Twenty four hours thereafter, radiolabelled thymidine (3H-TdR, 1.5 microCi g-1) was given by repeated injections during 24 h. An emergence of small, pyroninophilic ('tigroid') foci was observed at the second, fifth and eighth days after the proliferative stimulus. The focal putative precancerous cells presented a significant higher labelling index (L1) than the non-affected parenchymal cells for all exposure times. However, the labelling intensity decreased from the second to the eighth day after CCl4, suggesting a dilution of the radiolabelled DNA by repeated divisions within the foci. The nuclei of the same foci were analysed for DNA-content by feulgen microdensitometry on neighbouring sections. A gradual reduction of nuclear DNA-content was observed in 66% of the foci at the fifth day and in 100% of foci at the eight day, as compared to surrounding tissue and untreated animals, where labelling and DNA-content remain in the same ratio.


Br. J. Cancer (1989), 60, 827-833 © The Macmillan Press Ltd., 1989

### Cell population kinetics and ploidy rate of early focal lesions during hepatocarcinogenesis in the rat

Ph. Castelain[1], A. Deleener[1], M. Kirsch-Volders[1] & H. Barbason[2]

[1]*Laboratory for Anthropogenetics, VUB, Pleinlaan 2, 1050 Brussels, and* [2]*Tour de Pathologie B35, ULG, Sart Tilman, 4000 Liege, Belgium.*

**Summary** We have studied the changes in cell population kinetics and DNA-content of cycling parenchymal cells during the very early steps of rat hepatocarcinogenesis in Faber's protocol. Adult rats were initiated by a single dose of diethylnitrosamine (DENA, 200 mg $kg^{-1}$), followed 2 weeks later by a 2-week diet of 0.03% 2-acetylaminofluorene (2-AAF) as selection phase. In the middle of selectiòn time, a single necrogenic dose of carbon tetrachloride ($CCl_4$, 2 ml $kg^{-1}$) was administered by gavage. Twenty four hours thereafter, radiolabelled thymidine ($^3$H-TdR, 1.5 μCi $g^{-1}$) was given by repeated injections during 24 h. An emergence of small, pyroninophilic ('tigroid') foci was observed at the second, fifth and eighth days after the proliferative stimulus. The focal putative precancerous cells presented a significant higher labelling index (L1) than the non-affected parenchymal cells for all exposure times. However, the labelling intensity decreased from the second to the eighth day after $CCl_4$, suggesting a dilution of the radiolabelled DNA by repeated divisions within the foci. The nuclei of the same foci were analysed for DNA-content by feulgen microdensitometry on neighbouring sections. A gradual reduction of nuclear DNA-content was observed in 66% of the foci at the fifth day and in 100% of foci at the eight day, as compared to surrounding tissue and untreated animals, where labelling and DNA-content remain in the same ratio.

It is well established now that carcinogenesis is a multistep process (Bannasch *et al.*, 1980; Farber & Cameron, 1980; Farber & Sarma, 1987). In rat hepatocarcinogenesis, many models are available which generate preneoplastic lesions as foci and nodules, which are believed to be preferential sites for ultimate cancer development (Farber & Cameron, 1980). Some of these protocols allow a step-by-step analysis of these early preneoplastic foci and nodules, because of their synchroneous emergence (Farber *et al.*, 1976; Lans *et al.*, 1983). A lot of biochemical, enzymatic and/or genetic alterations were described in the past, but the problem is that none of these markers seems to persist until the final cancerous stages.

In previous works (Deleener *et al.*, 1987; Kirsch-Volders *et al.*, 1986) it was shown that nodular cells, induced by a triphasic protocol (initiation, selection, promotion) were predominantely diploid, in contrast to the mainly tetraploid cell population of a non-treated adult rat liver. These findings were also reported with several other carcinogenic regimens (Bassleer *et al.*, 1985; Godoy *et al.*, 1976; Inui *et al.*, 1971; Pugh & Goldfarb, 1978; Schwarze *et al.*, 1984; Styles *et al.*, 1985, 1987; Sargent *et al.*, 1989) and even in primary liver cancer of man (Saetar *et al.*, 1987). These downward shifts in ploidy level were observed by classical cytodensitometry (Inui *et al.*, 1971; Neal & Bulter, 1978), flow-cytometry of interphase cells (Schwarze *et al.*, 1984; Styles *et al.*, 1985) or by chromosome counting of dividing hepatocytes (Becker *et al.*, 1971).

In other studies, however, preneoplastic and cancerous lesions were not unequivocally diploid, but also showed a tetraploid pattern (Bassleer *et al.*, 1985; Kuo *et al.*, 1987; Mori *et al.*, 1982; Sarafoff *et al.*, 1986).

Besides the biological meaning of diploidisation, the question arises at what time and in which cells this phenomenon develops. Hitherto, many authors described the growth kinetics of hyperplastic and putative premalignant cell populations. In these works, emphasis was laid on the importance of cellular proliferation after initiation (Columbano *et al.*, 1981; Ying *et al.*, 1982) and on the effect of several carcinogens upon cell loss, repair mechanisms and the concommitant *de novo* DNA synthesis (Albert *et al.*, 1972; Bursch *et al.*, 1985; Yager & Potter, 1975). Moreover, it is generally accepted that there is a significant increase of DNA synthesis and mitotic activity in foci and nodules induced by carcinogens. The latter was demonstrated by combinations of enzyme histochemical techniques and histoautoradiography after chronic or pulse-labelling with radioactive precursors of DNA (Barbason *et al.*, 1983; Enomoto & Faber, 1982; Kitagawa & Sugano, 1973; Pugh & Goldfarb, 1978; Rabes & Szymkowiak, 1979; Rotstein *et al.*, 1984, 1986; Schulte-Hermann *et al.*, 1983). None of these works, however, reports the link between the proliferative activity of putative preneoplastic lesions and the ploidy of their nuclei.

In this study, we tried to follow the fate of the cycling cells during the selection phase of the biphasic protocol for hepatocarcinogenesis (i.e. period of focal growth). This was done by following the incorporation of a radioactive precursor for DNA on autoradiographs. In this way, cells were labelled which resist the cytotoxic effects of the 'selector' 2-AAF and of the necrogenic agents $CCl_4$. This period is interesting in the analysis because the resistent cells will grow out to possible preneoplastic lesions during this time. Histological changes were detected by classical Haematoxylin and Eosin staining and methyl green-pyronine staining; DNA-content was analysed on Feulgen-stained serial sections by microdensitometry.

### Materials and methods

### *Experimental protocol (Figure 1)*

*Initiation–selection (IS-$CCl_4$) protocol* Twenty male Wistar-R rats (IOPS AF) HAN, Iffa Credo, about 3 months old, were injected i.p. with a necrogenic dose of diethylnitrosamine (DENA) 200 mg $kg^{-1}$ i.s. 0.9% NaCl for initiation. Two weeks later a selection regimen was given, as described by Lans *et al.* (1983). A 0.03% solution of 2-acetylaminofluorene (2-AAF) was added to the UAR (04) basal diet. This regimen was given during 2 weeks. In the middle of this period, carbon tetrachloride ($CCl_4$) was administered by gavage at a dose of 2 ml $kg^{-1}$, diluted with an equal volume of corn oil. This serves as a proliferative stimulus for non-inhibited hepatocytes.

*$CCl_4$ protocol* As a comparison, 10 age-matched animals were treated only with $CCl_4$ at the same time-point as the rats treated in the IS protocol. No DENA or 2-AAF was given to them.

*Untreated controls* As a supplementary control, five rats receive a normal regimen, and will be referred further on as the 'untreated group'.

Correspondence: Ph. Castelain.
Received 26 September 1988; and in revised form 8 June 1989.

**Figure 1** Experimental protocol for rat hepatocarcinogenesis. Wistar R male rats about 250 g. DENA, diethylnitrosamine, 200 mg kg$^{-1}$; 2-AAF, 2-acetylaminofluorene, 0.03%; $CCl_4$, carbon tetrachloride, i.g. 2 ml kg$^{-1}$, 1:1 dil. in corn oil; T*, $^3$H-TdR, 1.5 $\mu$Cig$^{-1}$, 6 i.p. injections during 24 hours; NN, normal nourishment.

*Incorporation of radioactive thymidine*

*Initiation–selection protocol (IS-$CCl_4$)* In order to label a significant proliferating cell-fraction, a continuous 24-h incorporation of 6-$^3$H-TdR was performed as follows: the radiolabelled thymidine (1.5 $\mu$Ci g$^{-1}$, specific activity 5 Ci mmol$^{-1}$ diluted in a.d., Amersham Int) was injected i.p. four times at 6-h intervals from the 24th hour to the 48th hour after the $CCl_4$ administration. In this way, all cells passing through DNA synthesis in the time span of 24 h are labelled (assuming the mean S-phase duration of normal liver cells to be about 7 h) (Rabes & Szymkowiak, 1979; Barbason *et al.*, 1983). Radiotoxicity, caused by the $^3$H-TdR was unlikely. No significant lethalithy is expected, since each cell received no more than 3 $\mu$Ci g$^{-1}$ body weight in total (1.5 $\mu$Ci g$^{-1}$ at 6-h interval labels at a maximum twice in the same cell, since the mean $t_c$ is $\pm$40 h and the total $^3$H-TdR-administration lasts for 24 h).

All animals were treated in the same way. They were killed in groups of five respectively 2 days, 5 days, 8 days or 14 days after the $CCl_4$ administration. The animals of this experimental group are referred to as 'IS-2, 5 or 8d' in the text.

*Controls* The $CCl_4$ group and untreated group received no $^3$H-TdR. The 10 animals of the $CCl_4$ group were killed in groups of five respectively 2 days and 8 days after the necrogenic stimulus. The livers of these control animals were only used to analyse the DNA-content of hepatocytes.

*Histology*

After excision, pieces of liver were fixed in either 10% formalin or Carnoy solution (6:3:1 of absolute ethanol:chloroform:acetic acid) and embedded in paraffin.

Sections of 7 $\mu$m were cut serially and stained with Haematoxylin and Eosin (H&E), methyl green-pyronine (Unna-Brachet stain) (UB) and Feulgen. All stained slices were dehydrated in changes of graded ethanol and mounted with DPX (Fluka).

At least one section was processed for histoautoradiography. For this purpose, slides were coated with K5-emulsion (Ilford) by dipping and stored in the dark at 4°C for 1, 2, 3, 4 or 5 weeks. After 3 weeks, a plateau in the labelling intensity was reached (optimal labelling without too great background labelling). The slices were developed and post-stained with Unna-Brachet. Cells with five or more grains over their nucleus were considered as labelled (this was based on the analysis of the background level of the autoradiography, which was less than five grains per unit area ( = area of one nucleus).

For the Feulgen reaction, Carnoy-fixed slices were first hydrolysed under mild conditions (1 N HCl at room temperature for 17 h). This hydrolysis time was chosen because of the stability of the Feulgen stain at this time-point (hydrolysis curve not shown). After rinsing, Feulgen stain was performed during 1 h, and rinsed with saturation buffer during 10 min.

*Morphometry and cytodensitometry*

Early lesions were visualised with H&E and UB stain. The slices were projected on a drawing table; the lobes and early foci were drawn with an 16.5 times enlargement. The areas of the lobe and focal sections were measured on a HP 9874A digitizer.

The labelling index (LI), defined as the percentage of labelled nuclei in the total number of nuclei, was measured on a glarex projection screen, mounted upon a Zeiss microscope.

Using a magnification of 400 times, constant areas (370 $\mu$m) were randomly analysed.

The DNA-content of focal and non-focal nuclei was determined by Feulgen microdensitometry. For this purpose, pyroninophilic foci (as determined by the UB stain) were photographed with high magnification. The same lesions were relocated on a Feulgen-stained section. Densitometric measurements were performed on a computer-assisted image analysing device Magiscan 2A (Joyce-Loebl, GB) connected to a Zeiss photomicroscope III with a Bosch TV camera (TYK 9A, Chalnicon tube).

### Results

*Morphometric data on growth of hyperplastic foci*

Two days after IS-$CCl_4$, an important necrosis was observed, predominantly in centrolobular areas. These areas were characterised by karyorhexic, karyolytic and heteropycnotic nuclei. Eight days after IS-$CCl_4$ these degenerating cells are no longer observed and non-parenchymal oval cells appeared. After labelling with $^3$H-TdR, these necrotic cells were heavily labelled at 2 days after IS-$CCl_4$. This labelling disappeared after 8 days in these areas. The H&E, and especially the UB-staining revealed little, pyroninophilic foci ('tigroid' foci) from the second day after IS-$CCl_4$ on. They were characterised by cells with clusters of RNA in their cytoplasm and sometimes very prominent nucleoli, as previously reported (Bannasch *et al.*, 1985). The morphometric data, given in Table I, show that there was an increase of the volume and of the number of these foci in the course of the exposure time. From the second to the fifth day, the number of foci increases with a factor 2, and the volume remains somewhat constant. From the fifth to the eighth day, the volume is doubling, while the number of foci increases with a factor 3. The increase in the fraction of focal tissue (the total area of the foci as a percentage of the total liver section area) indicates that the growth of the foci exceeds by far the reparative growth of the rest of the liver parenchyma. The increase in number and in volume of the tigroid foci reaches a maximum at the eighth day after the IS-$CCl_4$ induction (mean values per treatment in Table I). However, after 14 days, it appears that there is a confluence of the pyroninophilic foci: they are not observable as single entities any more.

A similar observation was made in livers of animals which were treated with $CCl_4$ alone: also in this case, 2 days after $CCl_4$ small pyroninophilic foci were noticed, which persisted until the eighth day. No morphometric data were collected on this focal proliferation, since these foci were very small and not sharply delimited in the surrounding liver tissue.

*Proliferative activity of resistant cells*

In order to know which cells were cycling in the 24 h period immediately after the $CCl_4$ induction, the labelling index was determined in necrotic regions, normal cells and focal cells. The results are given in Table II.

Two days after $CCl_4$, there is a huge incorporation in the

**Table I** Data of relative focal area, fraction of focal tissue, and number of foci per unit area

| *Treatment* | *No.* | *Relative focal area (%)* | *s.d.* | *Fraction of focal tissue (per mille)* | *Number of foci per cm²* |
|---|---|---|---|---|---|
| 2 days | 1 | 6.32 | 1.28 | 0.40 | 5.70 |
| | 2 | 8.28 | 1.63 | 0.34 | 5.14 |
| | 3 | – | – | – | – |
| | 4 | – | – | – | – |
| | 5 | 16.50 | 4.51 | 2.16 | 10.38 |
| | Mean | 9.43 | 4.21 | 0.97 | 7.10 |
| 5 days | 1 | 8.31 | 2.85 | 0.67 | 13.43 |
| | 2 | 11.80 | 0.57 | 1.91 | 17.57 |
| | 3 | 14.70 | 1.00 | 3.17 | 22.13 |
| | 4 | – | – | – | – |
| | 5 | 8.68 | 3.59 | 0.31 | 4.81 |
| | Mean | 10.87 | 2.59 | 1.52 | 14.50 |
| 8 days | 1 | 19.48 | 3.68 | 5.35 | 26.51 |
| | 2 | 35.10 | 0.18 | 3.50 | 105.44 |
| | 3 | 26.90 | 3.78 | 5.10 | 59.78 |
| | 4 | 23.70 | 2.80 | 4.54 | 19.33 |
| | 5 | 16.20 | 1.61 | 3.17 | 20.74 |
| | Mean | 24.28 | 6.52 | 4.30 | 46.36 |

[a]Relative focal area is the mean area of the foci (in $\mu m^2$) divided by the total area of the lobe. [b]Fraction of focal tissue is the sum of the areas of all foci in a lobe divided by the total area of that lobe. The treatment is indicated by the number of days after the proliferative stimulus ($CCl_4$) in the IS protocol.

**Table II** Mean labelling index (L1 in %) of necrotic, normal and focal cells for different periods after the necrogenic stimulus (in the IS protocol)

| | | *Labelling index (%)* | | |
|---|---|---|---|---|
| *Treatment* | *No.* | *Necrotic zone* | *Non-affected parenchyma* | *Focal tissue* |
| 2 days | 1 | 30.6 | 4.4 | – |
| | 2 | 43.8 | 9.5 | 76.1 |
| | 3 | 36.6 | 8.4 | 71.9 |
| | 4 | 49.6 | 10.7 | – |
| | 5 | 47.6 | 12.2 | 77.3 |
| | Mean | 37.6 | 7.6 | 75.1 |
| 5 days | 1 | 24.1 | 12.6 | 73.1 |
| | 2 | 20.9 | 4.1 | 56.9 |
| | 3 | 38.7 | 10.1 | 81.6 |
| | 4 | 45.6 | 35.1 | – |
| | 5 | 24.3 | 9.1 | – |
| | Mean | 33.7 | 10.3 | 70.5 |
| 8 days | 1 | – | 10.7 | 50.3 |
| | 2 | – | 11.7 | 49.9 |
| | 3 | – | 9.4 | 39.6 |
| | 4 | – | 16.8 | 45.6 |
| | 5 | – | 2.7 | 49.9 |
| | Mean | – | 10.6 | 47.1 |
| 14 days | 1 | – | 4.8 | – |
| | 2 | – | 3.8 | – |
| | 3 | – | 1.6 | – |
| | 4 | – | 2.4 | – |
| | 5 | – | – | – |
| | Mean | – | 3.6 | – |

necrotic zones (38% LI). This LI decreases to 34% until the fifth day and decreases further to nearly 0% afterwards, indicating a massive cell loss. In the non-affected parenchyma, the LI increases somewhat from the second to the eighth day to 10.6% and decreases after the eighth day until the fourteenth day to 3.6%.

The foci show a very high LI compared to the surrounding parenchyma. They are particularly strongly labelled after the second day and after the fifth day, but between the fifth and the eighth day, the LI decreases to about 67% (compared to the value at 5 days). The relationship between the volume of a focus and its mean LI was investigated by calculating the correlation between them (Figure 2): a significant negative correlation coefficient of 0.76 was obtained.

**Figure 2** Correlation between the relative area of the foci (fraction of focal area) and the labelling index.

### *DNA content*

The nuclear DNA content was determined on serial sections after Feulgen staining and analysed with a computer-aided cytodensitometer. The histograms of DNA-content are given on Figure 3.

Figure 3 givens the distributions of DNA-content from hepatocytes of normal, perifocal and focal tissue, collected from the several animals submitted to the same treatment. As a general remark, it appears that there is a broad range in the C-value distribution, especially for the normal tissue. In the first group (2 days after IS-$CCl_4$) only one animal shows measurable foci (Figure 3). In this animal, no significant shift to lower C-values can be noted in the focal tissue, as compared to the normal parenchyma. Moreover, there is a significant difference between the distribution of this animal and the merged data from the other animals of the same group, indicating a strong interindividual variation.

On the fifth day after IS-$CCl_4$, the shift of the modal C-value is more clear. On the eighth day, the downward trend is confirmed. It appears that about 60% of the focal cells have a ploidy rate beneath 4C (compared to 36% on the fifth day and 14% on the second day).

There is no significant difference between the DNA-profiles of the untreated livers and these of the livers only treated by the $CCl_4$ (see Figure 3). If there is no initiation or selection, the mean result is a bimodal distribution round the 4C- and 8C-values These results were also obtained in 'focal' cells generated by the $CCl_4$ alone. This suggests that these foci are only cirrhotic lesions; it is known that a treatment with $CCl_4$ alone, even with this high dose, is not sufficient to induce hepatocarcinogenesis in rats.

In Figure 4, a survey of the C-values is given. The percentage C-value is given per treatment and per kind of tissue. The classes are defined here as the integrated part (in %) of the histograms in Figure 3: in order to define 2C, 4C and 8C fractions, two boundaries were created around the three modal C-values (see as an example the DNA profile of the untreated liver in Figure 5). For the data of the animals treated with the IS-protocol, a clearcut increase in the 2C fraction is visible. At the same time the 4C fraction and the 8C fraction decrease. A striking observation is that the decrease in the 8C fraction is the strongest, as compared to the decrease of the 4C fraction. This evolution is observed in the course of the treatment, as well as within the different type of tissues (non-focal, perifocal and focal).

That the reduction of nuclear ploidy rate really corresponds to a reduction of cellular ploidy rate is proven by the nuclearity analysis summarised in Table III. Of the focal and of the normal cells 95–97% are mononucleated.

### Discussion

### *Morphometric data on the growth of hyperplasic foci*

Between the second and the fifth days after $CCl_4$ the mean focal area is relatively constant while the number of foci per

**Figure 3** Merged histograms of proportion of cells in % (ordinate) with a given integrated optical density in arbitrary units (abscissa). The data are given per treatment (IS, treated wth DENA, 2-AAF and $CCl_4$ controls are only treated with $CCl_4$) and per kind of tissue (non-focal, perifocal and focal). The treatments and the kind of tissue are indicated above each histogram. The number of cells analysed is designated by *n*. The modal C-values (2C, 4C and 8C) are delimited by the vertical bars in the histograms.

unit area doubles, in parallel with the doubling of the fraction of focal tissue (Table I). From these data, it can be deduced that the increase in the total focal mass can be attributed mainly to the increase in the number of foci. At 8 days after $CCl_4$, there is a maximum in the fraction of focal tissue. This fraction has increased by a factor of 3 (compared to the value at 5 days). However, the increase cannot be related only to the increase in number, but also to an increase in volume of the existing foci. Between the fifth and eighth days there is an increase in the mean diameter of the tigroid foci. The larger the diameter of a focus, the greater is the probability of seeing it in a random section.

**Figure 4** Summarising column-chart of ploidy level (abscissa) for the different treatments (2 days, 5 days and 8 days) and for the different tissues. Percentages (ordinate) are calculated by the integration of several classes in the histograms in Figure 3.

**Figure 5** DNA profile of untreated liver, to illustrate the way by which C-fractions were calculated. The grouping of classes is shown in abscissa. The modal C-values are given by the dotted lines.

**Table III** Frequency of binucleated cells in normal and in focal tissue (in %)

| | | *% binucleated cells* | |
|---|---|---|---|
| *Treatment* | *No.* | *Normal cells* | *Focal cells* |
| 2 days | 1 | 3.4 | – |
| | 2 | 7.1 | – |
| | 3 | 4.4 | – |
| | 4 | 6.2 | – |
| | 5 | 8.1 | – |
| 5 days | 1 | 3.8 | – |
| | 2 | 2.2 | – |
| | 3 | 2.8 | – |
| | 4 | 3.2 | – |
| | 5 | 2.4 | – |
| 8 days | 1 | 3.3 | 5.2 |
| | 2 | 5.6 | 3.3 |
| | 3 | 3.4 | 4.7 |
| | 4 | 2.6 | – |
| | 5 | 2.6 | – |
| 14 days | 1 | 3.2 | – |
| | 2 | 4.1 | – |
| | 3 | 4.2 | – |
| | 4 | 4.6 | – |
| | 5 | 5.6 | – |

### *Autoradiographic data on proliferating cells*

The massive incorporation of $^3$H-TdR at 2 days after $CCl_4$ may be an indication of DNA repair synthesis as a regenerative response against the xenobiotic (Yager *et al.*, 1975). This is followed by a massive cellular loss, for virtually no labelling can be detected in the centrolobular regions from the eighth day onwards. On the other hand, it cannot be excluded that a part of the labelling can be ascribed to the proliferation of non-parenchymal cells such as endothelial cells (Tatematsu *et al.*, 1984) or Kupfercells (Bouwens *et al.*, 1986). However, almost no labelled non-parenchymal cells are observed later on in the liver. For that reason, the non-parenchymal cells represent only a minority among the labelled cells from the centrolobular zone. Massive re-utilisation of the $^3$H-TdR is unlikely, the biological half-life of the radioactive precursor being only 1 h.

The experiment further indicates that selection with 2-AAF does not produce a complete inhibition of the DNA synthetic activity as depicted by the hypothesis of Solt *et al.* (1977), since 3.6–10.6% of the cell population is cycling in the non-focal parenchyma.

When the data of LI of tigroid foci are considered (Table II), it appears that there is a reduction by a factor 1.6 between the second and the eighth days after $CCl_4$. This reduction of LI can be the consequence of a cellular loss, or of cellular division of at least a part of the focal cell population.

When this finding is compared to the morphometric data of focal growth discussed above, it is clear that the latter is more likely to occur. In the timespan of 6 days the fraction of focal tissue increased by a factor of 4.4, which implies two rounds of cell division.

Another argument for their intense mitotic activity is found in the inverse relationship between the total volume of the foci and their mean LI. Figure 2 shows the high correlation between the volume of the focus and the mean LI. This can be explained by the dilution of the $^3$H-TdR in the focus by repeated divisions. This does not exclude the possibility that there is DNA repair in the foci, but in this case we would see a strong labelling in the focal cells, even 14 days after the $CCl_4$ induction. This phenomenon has not been observed here.

Many data in the literature evoke the presence of highly proliferating cells in preneoplastic lesions in other protocols (Pugh & Goldfarb, 1978; Enomoto & Farber, 1982; Garcea *et al.*, 1987).

*Cytodensitometric data on DNA content*

The broad spread in the DNA distribution of the cell populations (Figure 3) can partly be explained by the fact that the analysis of DNA content was made on histological sections. In contrast to flow-cytometric measurements, it is more difficult to distinguish between the different maxima of 2C, 4C, etc. In our system, however, the produced foci are so small that a liver perfusion before flow-cytometry would produce a mixture of focal and non-focal cells. Consequently, the DNA-profiles of these entities would be masked by those of the non-affected parenchymal cells. A second explanation for the broad histograms is the presence of a significant S-phase fraction in the treated livers.

The reduction in DNA content of focal cells is only clear in the period when the number and fraction of foci is at its highest, i.e. 8 days after the $CCl_4$ induction. Five days after $CCl_4$, this trend is not so clear. After 14 days, the diploid foci are not visible as separate units any more. This may be the consequence of an intensive proliferation of these foci and of the rest of the parenchyma (the latter can occur now because there is no mito-inhibition by the 2-AAF any longer), so that they dissipate in the surrounding parenchyma. It is striking that perifocal nuclei show intermediate values between those of the normal and the focal tissue. However, this may be the consequence of a technical artefact. It cannot be excluded that the observed perifocal cell population is a mixture of focal and normal cells, because of the difficulty in delineating perfectly the foci on serial sections.

It must further be stressed that there is an important interindividual variation between animals receiving the same treatment, so that care must be taken with the summing up of the individual data. In spite of this variation a trend in the reduction to lower C-values can be seen in the merged data. This means that the differences between different tissues in the same animal are always significantly greater than the differences between different animals. Emphasis must also be laid on the fact that these C-values do not correspond to actual ploidy values. A 4C value, for example, can be a 2N-nucleus in $G_2$ or a 4N-nucleus in $G_1$. Without chromosome counting it is impossible to discriminate between them on that basis alone.

The question remains of by which mechanism preneoplastic focal cells preferentially contain nuclei in the 2C DNA-range. A theoretically conceivable hypothesis resides in the fact that there could exist a population of hepatocytes, delayed or blocked in $G_2$ phase (Bassleer *et al.*, 1985). This phenomenon was reported in skin (Gelfant, 1977) and recently suggested in adult liver (Daoust, 1987). In this way, an appropriate stimulus could drive blocked 'tetraploid' (4C) nuclei into mitosis within a few hours and give rise to diploid hepatocytes in $G_0$. This mechanism is unlikely to occur in our model, because of the quasi-absence of unlabelled foci, at least in the early stages.

A second possibility is based upon the fact that $CCl_4$ causes a massive centrolubular necrosis in the liver, because of the metabolic zonation in this organ (Jungermann, 1986). This could suggest that the remaining periportal parenchymal cells would be the preferential candidates for liver regeneration (Fabrikant, 1968; Grisham, 1962). Since these periportal cells are mostly diploid (Sulkin, 1943) it could be expected that emerging cycling cells would have this ploidy level. However, early foci 5 days after IS-$CCl_4$ are not exclusively diploid; foci of higher ploidy rate also emerge at that time. Moreover, it was reported earlier that there was no preferential lobular distribution of early foci (Solt *et al.*, 1977). Thus, selective necrosis of tetraploid cells cannot be considered as the only mechanism of diploidisation. Rather, even tetraploid focal cells can emerge, together with the diploid ones.

This last remark holds also for the hepatocytes which were only hit by the $CCl_4$. The fraction of 2C cells which are proliferating is as great as the 4C fraction. This confirms the hypothesis which states that the outgrowth of 2C cells (in the IS-protocol) is only due to the general observation that the mitosis of diploid cells occurs more easily than the mitosis of tetraploid cells. There must be another factor, linked to the effects of initiation and selection, which makes diploid cells more likely to evolve to precancerous states than tetraploid ones.

Whether the process of diploidisation is a general process, or only confined to certain protocols, is an unsolved problem. It cannot be excluded that, in spite of the fact that ploidy reduction has been observed in many carcinogenic conditions, this phenomenon is the result of a co-selection, only linked to experimental set-up. In order to ascertain whether the polyploidisation-block described above is a fundamental process, or a necessary condition for cancer development, more hepatocarcinogenic protocols should be analysed for this parameter.

Nevertheless, it is possible that mainly diploid foci remain at later stages of the hepatocarcinogenic process, because of a physiological advantage in their favour. Previous work (Deleener *et al.*, 1987) reported diploidisation of early preneoplastic nodules in the I-S-P protocol (Lans *et al.*, 1983). It was shown that this phenomenon was not transient, but lasted for months after the onet of promotion. However, it must be stressed that if foci and nodules are preferentially diploid, this diploidisation is not necessarily a characteristic of hepatic carcinoma. This means that the process of diploidisation in the liver is linked to the genetic instability of preneoplastic lesions, rather than to the cancer phenotype.

One of the possibilities is that the diploid cell population may be at higher risk for further carcinogenic alterations, because some cancer phenotypes might be expressed more easy in a diploid than in a tetraploid genome (on the assumption that these cancer phenotypes are the result of recessive traits).

The authors wish to thank Dr H. Taper (UCL) for help in evaluating the histological alterations, Dr J. de Gerlache (UCL) for supplying the carcinogenic diets, and M. Vanmechelen and Fr. Raymaekers for their technical assistance.

### References

ALBERT, R.E., BURNS, F.J., BILGER, L., GARDNER, D. & TROLL, W. (1972). Cell loss and proliferation induced by *N*-2-fluorenylacetamide in the rat liver in relation to hepatoma induction. *Cancer Res.*, **32**, 2172.

BANNASCH, P., MAYER, D. & HACKER, H.J. (1980). Hepatocellular glycogenosis and hepatocarcinogenesis. *Biochim. Biophys. Acta*, **605**, 217.

BANNASCH, P., BENNER, U., ENZMANN, H. & HACKER, H.J. (1985). Tigroid cell foci and neoplastic nodules in the liver of rats treated with a single dose of aflatoxin B1. *Carcinogenesis*, **6**, 1641.

BARBASON, H., RASSENFOSSE, C. & BETZ, E.M. (1983). Promotion mechanisms of phenobarbital and partial hepatectomy in DENA hepatocarcinogenesis cell kinetics effects. *Br. J. Cancer*, **47**, 517.

BASSLEER, R., DE PAERMENTIER, F. & BARBASON, H. (1985). Effects of diethylnitrosamine on deoxyribonucleic acid content and nucleoli in rat hepatocytes. A precancer state analysis. *Mol. Physiol.*, **7**, 78

BECKER, F.F., FOX, R.A., KLEIN, K.M. & WOLMAN, S.R. (1971). Chromosome patterns in rat hepatocytes during *N*-2-fluorenylacetamide carcinogenesis. *J. Natl Cancer Inst.*, **46**, 1261.

BOUWENS, L., BAEKELAND, M. & WISSE, E. (1986). Cytokinetic analysis of the expanding Kupffer-cell population in rat liver. *Cell Tissue Kinetics*, **19**, 217.

BURSCH, W., TAPER, H.S., LAUER, B. & SCHULTE-HERMANN, R. (1985). Quantitative histological and histochemical studies on the occurance and stages of controlled cell death (apoptosis) during regression of rat liver hyperplasia. *Virchows Arch. (Cell Pathol.)*, **50**, 153.

COLUMBANO, A., RAJALAKSHIMI, S. & SARMA, D.S.R. (1981). Requirement of cell proliferation for the initiation of liver carcinogenesis as assayed by three different procedures. *Cancer Res.*, **41**, 2079.

DAOUST, R. (1987). The passage of G2 hepatocytes into mitosis during fasting. *Chem.-Biol. Interact.*, **62**, 99.

DELEENER, A., CASTELAIN, PH., PREAT, V., DE GERLACHE, J., ALAXANDRE, H. & KIRSCH-VOLDERS, M. (1987). Changes in nucleolar transcriptional activity and nuclear DNA content during the first steps of rat hepatocarcinogenesis. *Carcinogenesis*, **8**, 195.

ENOMOTO, K. & FARBER, E. (1982). Kinetics of phenotype maturation of remodeling of hyperplastic nodules during liver carcinogenesis. *Cancer Res.*, **42**, 2330.

FABRIKANT, J.I. (1968). The kinetics of cellular proliferation in regenerating liver. *J. Cell Biol.*, **36**, 551.

FARBER, E. & CAMERON, R. (1980). The sequential analysis of cancer development. *Adv. Cancer. Res.*, **35**, 125.

FARBER, E., PARKER, S. & GRUENSTEIN, M. (1976). The resistence of putative premalignant liver cell populations, hyperplastic nodules, to the acute cytotoxic effects of some hepatocarcinogens. *Cancer Res.*, **36**, 3879.

FARBER, E. & SARMA, D.S.R. (1987). Biology of disease. Hepatocarcinogenesis: a dynamic cellular perspective. *Lab. Invest.*, **56**, 4.

GARCEA, R., PASCALE, R., DAINO, L. & 6 others (1987). Variations of ornithine decarboxylase activity and S-adenosyl-L-methionine and 5'-methylthioadenosine contents during the development of diethylnitrosamine-induced liver hyperplastic nodules and hepatocellular carcinoma. *Carcinogenesis*, **8**, 653.

GELFANT, S. (1977). A new concept of tissue and tumour cell proliferation. *Cancer Res.*, **37**, 3845.

GODOY, H.M., JUDAH, D.J., ARORA, H.L., NEAL, G.E. & JONES, G. (1976). The effect of prolonged feeding with aflatoxin B1 on adult rat liver. *Cancer Res.*, **36**, 2399.

GRISHAM, J.W. (1962). A morphologic study of deoxyribonucleic acid synthesis and cell proliferation in regenerating rat liver; autoradiography with thymidine-H3. *Cancer Res.*, **22**, 842.

INUI, N., TAKAYAMA, S. & KUWABARA, S. (1971). DNA measurements on cell nucleus of normal liver, adenoma, and hepatoma in mice: histologic features. *J. Natl Cancer Inst.*, **47**, 47.

JUNGERMANN, K. (1986). Functional heterogeneity of periportal and perivenous hepatocytes. *Enzyme*, **35**, 161.

KITAGAWA, T. & SUGANO, H. (1973). Combined enzyme histochemical and radioautographic studies on areas of hyperplasia in the liver of rats fed N-2-fluorenylacetamide. *Cancer Res.*, **33**, 2993.

KIRSCH-VOLDERS, M., DELEENER, A. & CASTELAIN, PH. (1986). Initiation and promotion: mechanisms of action and implication for risk estimation. Proceedings 11th Symposium Primary Cancer Prevention.

KUO, S.-H., SHEU, J.-C., CHEN, D.-S., SUNG, J.-L., LIN, C.-C. & HSU, H.-C. (1987). Cytophotometric measurements of nuclear DNA content in hepatocellular carcinomas. *Hepatology*, **7**, 330.

LANS, M., DE GERLACHE, J., TAPER, H.S., PREAT, V. & ROBERFROID, B.M. (1983). Phenobarbital as a promotor in the initiation/selection process of experimental hepatocarcinogenesis. *Carcinogenesis*, **4**, 141.

MORI, H., TANAKA, T., SUGIE, S., TAKAHASHI, S. & WILLIAMS, G.M. (1982). DNA content of liver cell nuclei of N-2-fluorenylacetamide-induced altered foci and neoplasms in rats and human hyperplastic foci. *J. Natl Cancer Inst.*, **69**, 1277.

NEAL, G.E. & BUTLER, W.H. (1978). A comparison of the changes induced in rat liver by feeding low levels of aflatoxin B1 or an azo dye. *Br. J. Cancer*, **37**, 55.

PUGH, T.D. & GOLDFARB, S. (1978). Quantitative histochemical and autoradiographic studies of hepatocarcinogenesis in rats fed 2-acetylaminofluorene followed by phenobarbital. *Cancer Res.*, **38**, 4450.

RABES, H.M. & SZYMKOWAIK, R. (1979). Cell kinetics of hepatocytes during the preneoplastic period of diethylnitrosamine-induced liver carcinogenesis. *Cancer Res.*, **39**, 1298.

ROTSTEIN, J., MACDONALD, P.D.M., RABES, H.M. & FARBER, E. (1984). Cell cycle kinetics of rat hepatocytes in early putative preneoplastic lesions in hepatocarcinogenesis. *Cancer Res.*, **44**, 2913.

ROTSTEIN, J., SARMA, D.S.R. & FARBER, E. (1986). Sequential alterations in growth control and cell dynamics of rat hepatocytes in early precancerous steps in hepatocarcinogenesis. *Cancer Res.*, **46**, 2377.

SAETER, G., SCHWARZE, P.E., OUS, S. & 4 others (1987). Reduced hepatocellular polyploidization is a common feature in experimental and human liver carcinogenesis. Abstract European Meeting on Experimental Hepatocarcinogenesis, 27–30 May, Spa, Belgium.

SARAFOFF, M., RABES, H.M. & DÖRMER, P. (1986). Correlations between ploidy and initiation probably determined by DNA cytophotometry in individual altered hepatic foci. *Carcinogenesis*, **7**, 1191.

SARGENT, L., XU, Y., SATTLER, G.L., MEISSNER, L. & PITOT, H. (1989). Ploidy and karyotype of hepatocytes isolated from enzyme-altered foci in two different protocols of multistage hepatocarcinogenesis in the rat. *Carcinogenesis*, **10**, 387.

SCHULTE-HERMANN, R., TIMMERMANN-TROSIENER, I. & SCHUPPLER, J. (1983). Promotion of spontaneous preneoplastic cells in rat liver as a possible explanation of tumour production by non-mutagenic compounds. *Cancer Res.*, **43**, 839.

SCHWARZE, P.E., PETTERSEN, E.O., SHOAIB, M.C, & SEGLEN, P.O. (1984). Emergence of a population of small, diploid hepatocytes during hepatocarcinogenesis. *Carcinogenesis*, **5**, 1267.

SOLT, D.B., MEDLINE, A. & FARBER, E. (1977). A rapid emergence of carcinogen-induced hyperplastic lesions in a new model for the sequential analysis of liver carcinogenesis. *Am. J. Pathol.*, **88**, 595.

STYLES, J., ELLIOT, B.M., LEFEVRE, P.A. & 4 others (1985). Irreversible depression in the ratio of tetraploid:diploid liver nuclei in rats treated with 3'-methyl-4-dimethylaminoazobenzene (3'M). *Carcinogenesis*, **6**, 21.

STYLES, J.A., KELLY, M. & ELCOMBE, C.R. (1987). A cytological comparison between regeneration, hyperplasia and early neoplasi in the rat liver. *Carcinogenesis*, **8**, 391.

SULKIN, N.M. (1943). A study of nucleus in normal and hyperplastic liver of the rat. *Am. J. Anat.*, **37**, 107.

TATEMATSU, M., HO, R.H., KAKU, T., EKEM, J.K. & FARBER, E. (1984). Studies on the proliferation and fate of oval cells in the liver of rats treated with 2-acetylaminofluorene and partial hepatectomy. *Am. J. Pathol.*, **114**, 418.

YAGER, J.D. & POTTER, V.R. (1975). A comparison of the effects of 3'-methyl-4-dimethylaminobenzene, 2-methyl-4-dimethylaminobenzene and 2-acetylaminofluorene on rat liver DNA stability and new synthesis. *Cancer Res.*, **35**, 1225.

YING, T.S., ENOMOTO, K., SARMA, D.S.R. & FARBER, E. (1982). Effects of delays in the cell cycle on the induction of preneoplasic and neoplasic lesions in rat liver by 1,2-dimethylhydrazine. *Cancer Res.*, **4**, 876.